# Coping with centriole loss: pericentriolar material maintenance after centriole degeneration

**DOI:** 10.1038/s42003-021-02243-6

**Published:** 2021-06-09

**Authors:** Carla M. C. Abreu, Tiago J. Dantas

**Affiliations:** 1 i3S - Instituto de Investigação e Inovação em Saúde, Universidade do Porto, Porto, Portugal; 2 IBMC - Instituto de Biologia Molecular e Celular, Universidade do Porto, Porto, Portugal

## Abstract

Shortly after the onset of ciliogenesis in *Caenorhabditis elegans* sensory neurons, the centrioles/basal bodies undergo degeneration. The fate of the pericentriolar material (PCM) that was associated with those centrioles has, however, remained unknown. Two recent studies by the Dammermann and the Feldman groups now show that not only does the PCM persist at the ciliary base, it also continues to assemble in the absence of canonical centrioles. Importantly, these neuronal centrosomes retain the ability to function as the cell’s main microtubule-organizing center and support ciliary function.

The centrosome is the main microtubule-organizing center (MTOC) in animal cells. This function is made possible through the assembly of a dense network of pericentriolar material (PCM) that nucleates and anchors microtubules during both interphase and mitosis. This PCM mesh surrounds a pair of barrel-shaped centrioles, which are microtubule-based structures with nine-fold symmetry, positioned at the core of centrosomes. While the main function of centrioles is to nucleate cilia, they have also been shown to serve as hubs to concentrate and maintain the centrosomal PCM.

Under certain circumstances, such as during oogenesis, centrioles and centrosomes undergo a process of elimination, in which the PCM dissipates and the centrioles are disintegrated or extruded (briefly reviewed in^1^). This complex mechanism of elimination serves in part to ensure that a newly fertilized zygote starts dividing with the correct number of centrioles/centrosomes. Interestingly, in ciliated sensory neurons of *Caenorhabditis elegans*, centrioles/basal bodies undergo a somewhat similar process known as centriole degeneration, although the reasons for its occurrence are far less clear.

The process of centriole degeneration in sensory neurons is known to start shortly after the initiation of ciliogenesis at the 1.5-fold stage of embryonic development^2,3^. This enigmatic process involves the loss of the central tube and most centriolar proteins, with the exception of HYLS-1 (HYLS1 in humans), which contributes to the initiation of intraflagellar transport (IFT) at the ciliary base. Interestingly, the outer wall of centriolar microtubules becomes flared (wider at the proximal end), but persists throughout development^2,3^. Whether the centrosomal PCM of these degenerated centrioles retains function or also disintegrates has remained unknown.

Two recent studies^4,5^ from the Dammermann and the Feldman groups revealed that even after centriole degeneration, the centrosome persists at the ciliary base in sensory neurons (Fig. 1). In fact, they showed that the PCM of this centrosome continues to incorporate the scaffolding proteins SPD-5 and PCMD-1, which are the closest orthologs of CDK5RAP2 and Pericentrin, respectively. Using 3D-SIM (Structured Illumination Microscopy), the Feldman group also nicely showed that both of these PCM proteins are able to infiltrate the proximal end of the ciliary axoneme, reaching into the transition zone region^5^ (Fig. 1). Importantly, both studies revealed that the acentriolar PCM continues to recruit γ-tubulin and other elements of the γ-tubulin ring complex (γ-TuRC) to nucleate and anchor microtubules, thus allowing for the centrosome to maintain its function as a MTOC. Consistent with this result, the authors showed that the microtubule network in these ciliated sensory neurons is highly polarized, emanating from the centrosome/MTOC at the dendritic tip towards the cell body^4,5^ (Fig. 1).

**Fig. 1:**
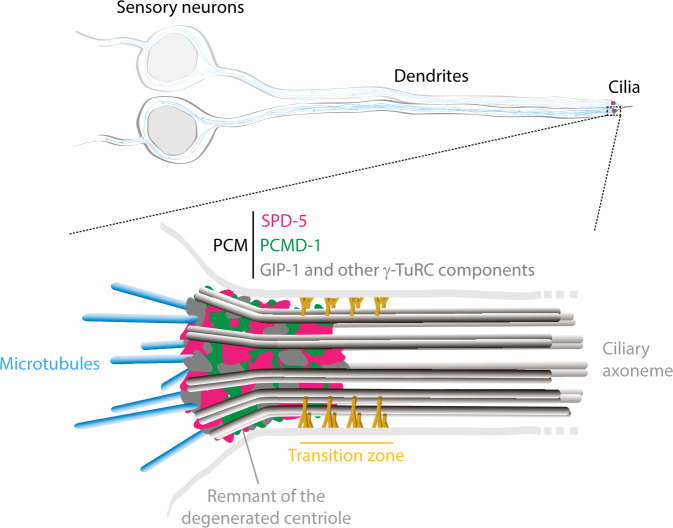
A matrix of SPD-5 and PCMD-1 scaffolding proteins form the PCM of centrosomes in *C. elegans* sensory neurons. This centrosomal PCM persists after centriole degeneration and continues to anchor γ-TuRCs to maintain the centrosome’s MTOC function.

Both groups were also able to shed light into the mechanisms regulating PCM assembly in these acentriolar centrosomes. They identified an X-box motif within the promoter region of *spd-5* that is responsible for driving the expression of this gene in ciliated sensory neurons by recruiting DAF-19 (a RFX-like transcription factor known to promote ciliogenesis). Then, using a cell type-specific degron to deplete SPD-5 or PCMD-1 in sensory neurons, the authors revealed that the assembly of these scaffolding proteins into the PCM is at least partially interdependent^4,5^. This finding led the Dammermann group to propose that SPD-5 and PCMD-1 synergize in a positive feedback loop to maintain the PCM in the absence of canonical centrioles.

In addition, the authors of both studies showed that the MTOC function of the centriole-less PCM at the ciliary base relies on the presence of SPD-5, which also anchors γ-TuRCs and microtubules to canonical centrosomes in the early embryo. In contrast, the mitotic PCM regulators PLK-1 and AIR-1 (orthologs of PLK1 and Aurora A, respectively) were dispensable for the integrity of the PCM in sensory neurons^4,5^. This was a striking finding for the authors as these kinases are well known to be critical for PCM maintenance and expansion in mitosis.

The Dammermann group went on to show that the presence of the PCM is not affected by the complete loss of the transition zone, the destabilization of the centriole remnant (*hyls*-*1* disruption), or even by blocking the entire ciliogenesis program (*daf-19* disruption)^4^.

Finally, although the impact of PCMD-1 loss in cilia differed in these studies, both groups showed that SPD-5 degradation clearly leads to significant defects in neuronal morphogenesis and in cilium assembly/maintenance^4,5^. The ciliary defects found by the authors could be at least in part explained by the disruption of microtubule-dependent transport of important ciliary factors towards the centrosome.

Altogether, these studies nicely showed that PCM can exist independently of centrioles in *C. elegans* and open a new avenue of research into the mechanisms of acentriolar PCM regulation. These studies also reinforce the concept that the PCM and MTOC function of centrosomes are important for ciliogenesis.

Given the association of many centrosomal and ciliary components with developmental disorders such as microcephaly and diverse ciliopathies, it is crucial to continue to dissect the biology of centrosomes and cilia. To underline the significance of research on these topics, we are currently organizing a special issue and inviting submissions on related topics at *Communications Biology*^6^.

## References

[1] Schoborg TA, Rusan NM (2016). Taking centrioles to the elimination round. Dev Cell.

[2] Nechipurenko, I. V., Berciu, C., Sengupta, P. & Nicastro, D. Centriolar remodeling underlies basal body maturation during ciliogenesis in Caenorhabditis elegans. *Elife*10.7554/eLife.25686 (2017). 10.7554/eLife.25686 PMC5392363 28411364

[3] Serwas D, Su TY, Roessler M, Wang S, Dammermann A (2017). Centrioles initiate cilia assembly but are dispensable for maturation and maintenance in *C. elegans*. J. Cell Biol..

[4] Garbrecht, J., Laos, T., Holzer, E., Dillinger, M. & Dammermann, A. An acentriolar centrosome at the *C. elegans* ciliary base. *Curr. Biol*. 10.1016/j.cub.2021.03.023 (2021). 10.1016/j.cub.2021.03.023 33798427

[5] Magescas, J., Eskinazi, S., Tran, M. V. & Feldman, J. L. Centriole-less pericentriolar material serves as a microtubule organizing center at the base of *C. elegans* sensory cilia. *Curr. Biol*. 10.1016/j.cub.2021.03.022 (2021). 10.1016/j.cub.2021.03.022 PMC8277230 33798428

[6] Dantas TJ (2020). Centrosomes and cilia: always at the center of the action. Commun. Biol..

